# IL-21 Restricts Virus-driven Treg Cell Expansion in Chronic LCMV Infection

**DOI:** 10.1371/journal.ppat.1003362

**Published:** 2013-05-16

**Authors:** Iwana Schmitz, Christoph Schneider, Anja Fröhlich, Helge Frebel, Daniel Christ, Warren J. Leonard, Tim Sparwasser, Annette Oxenius, Stefan Freigang, Manfred Kopf

**Affiliations:** 1 Molecular Biomedicine, Institute of Molecular Health Sciences, Department of Biology, ETH Zurich, Zurich, Switzerland; 2 Institute of Microbiology, Department of Biology, ETH Zurich, Zurich, Switzerland; 3 Garvan Institute of Medical Research, Darlinghurst, Sydney, Australia; 4 The University of New South Wales, Faculty of Medicine, St Vincent's Clinical School, Darlinghurst, Sydney, Australia; 5 Laboratory of Molecular Immunology and the Immunology Center, National Heart, Lung, and Blood Institute, National Institutes of Health, Bethesda, Maryland, United States of America; 6 Institute of Infection Immunology, TWINCORE/Centre for Experimental and Clinical Infection Research, Hannover, Germany; University of California, San Diego, United States of America

## Abstract

Foxp3^+^ regulatory T (Treg) cells are essential for the maintenance of immune homeostasis and tolerance. During viral infections, Treg cells can limit the immunopathology resulting from excessive inflammation, yet potentially inhibit effective antiviral T cell responses and promote virus persistence. We report here that the fast-replicating LCMV strain Docile triggers a massive expansion of the Treg population that directly correlates with the size of the virus inoculum and its tendency to establish a chronic, persistent infection. This Treg cell proliferation was greatly enhanced in IL-21R^−/−^ mice and depletion of Treg cells partially rescued defective CD8^+^ T cell cytokine responses and improved viral clearance in some but not all organs. Notably, IL-21 inhibited Treg cell expansion in a cell intrinsic manner. Moreover, experimental augmentation of Treg cells driven by injection of IL-2/anti-IL-2 immune complexes drastically impaired the functionality of the antiviral T cell response and impeded virus clearance. As a consequence, mice became highly susceptible to chronic infection following exposure to low virus doses. These findings reveal virus-driven Treg cell proliferation as potential evasion strategy that facilitates T cell exhaustion and virus persistence. Furthermore, they suggest that besides its primary function as a direct survival signal for antiviral CD8^+^ T cells during chronic infections, IL-21 may also indirectly promote CD8^+^ T cell poly-functionality by restricting the suppressive activity of infection-induced Treg cells.

## Author Summary

T cell exhaustion represents a state of T cell dysfunction associated with clinically relevant diseases, such as persistent viral infections or cancer. Although the molecular signature of exhausted T cells has been characterized in detail at the functional and transcriptional level, the immunological mechanisms that lead to T cell exhaustion during chronic infections remain poorly understood. Our present study reports two major findings that illustrate a pathway that contributes to T cell exhaustion during viral infection, and indicate its modulation by both, the pathogen and the host. First, we show that a persistence-inducing virus triggers the massive proliferation of Foxp3^+^ regulatory T (Treg) cells and demonstrate the potential of Treg cells to promote T cell exhaustion and chronic infection. Second, we identify IL-21 as a crucial host factor that antagonizes this virus-driven expansion of the Treg population in a cell intrinsic manner independent of IL-2. Thus, in addition to its known pre-dominant direct positive effects on antiviral T cells, IL-21 can also alleviate the suppressive activity of Treg cells. Together, these results suggest enhanced Treg cell responses as a mechanism of immune evasion that could be therapeutically targeted with IL-21.

## Introduction

The immune system has to efficiently eliminate pathogens but simultaneously needs to avoid the potential self-damage and immunopathology caused by excessive immune activation. Therefore, a tight regulation of immune responses is critical for host survival. The subset of CD4^+^CD25^+^ regulatory T (Treg) cells exerts key negative regulatory mechanisms of the immune system that prevent autoimmunity and T cell mediated inflammatory disease [1]. Treg cells are best defined by expression of the signature transcription factor forkhead box P3, Foxp3 [2], [3], [4], [5], [6], [7]. Their fundamental role in the maintenance of immune homeostasis and tolerance is well established [8], [9], [10] and unambiguously demonstrated by the severe multi-organ autoimmune disease, allergy and inflammatory bowel disease that develops in Foxp3-deficient mice or patients with immune dysregulation, polyendocrinopathy, enteropathy, X-linked (IPEX) syndrome [3], [11], [12], [13]. However, the relevance of Treg cell responses for shaping adaptive immunity against pathogens, in particular in the context of chronic infections, refigmains much less understood.

Treg cells potentially have both beneficial and adverse effects on disease outcomes during viral infections. By dampening effector immune responses, Treg cell responses mitigate immunopathology resulting from exaggerated inflammation and tissue destruction during acute [14], [15], [16], [17], or protracted infections [18], [19], [20], [21], [22]. In addition, Treg cells have been shown to support antiviral immunity by modulating T cell migration to the site of infection [15], [23]. Conversely, Treg cells were shown to suppress CD8^+^ T cell responses in some infections [21], [24], which may prevent immunopathology, but hampers effective pathogen control and ultimately promotes persistent infection [18], [21], [25], [26]. Thus, while Treg cells favorably influence pathogen clearance in many acute infections [14], [15], [16], [23], they seem to negatively regulate CD8^+^ T cell responses during chronic infections [18], [19], [20], [24], [26]. Furthermore, elevated numbers of Treg cells have also been associated with persistent viral infections in humans [27], [28], [29]. However, to date little is known as to whether Treg cell activation represents a mechanism of immune evasion that facilitates persistence, or about host factors that might regulate Treg cell responses during chronic viral infection.

Many characteristics of persistent viral infections in humans, such as HIV, HCV, or HBV, are also observed during chronic infection of mice with the arenavirus lymphocytic choriomeningitis virus (LCMV). Accordingly, murine models of chronic LCMV infection have been extensively studied and have provided key insights into the molecular mechanisms leading to virus persistence and the associated modulation of adaptive immunity in face of persistent infection. For example, infection with a high dose of the fast-replicating strain LCMV Docile (or Armstrong clone 13) results in virus persistence that is accompanied by a progressive functional impairment and – for some specificities – even leads to deletion of the virus-specific CD8^+^ T cell response, termed T cell “exhaustion” [30]. Exhausted antiviral T cells gradually lose their capacity to produce antiviral cytokines and to proliferate *ex vivo* [31], [32], [33]. Similar states of T cell exhaustion have been demonstrated in patients with chronic HIV or HCV infections [34]. CD8^+^ T cell exhaustion directly correlates with the antigen load, i.e. is enhanced during prolonged or high viral replication. Furthermore, CD8^+^ T cell exhaustion is also influenced by the sustained expression of several inhibitory receptors [35] and immunomodulatory cytokines, such as IL-10 [36], [37] and TGFβ [38]. Moreover, it can be aggravated by the loss of help or essential cytokines provided by CD4^+^ T cells. In particular, we and others [39], [40], [41] have recently identified the pro-inflammatory cytokine IL-21 as an essential CD4^+^ T cell-produced factor that prevents CD8^+^ T cell exhaustion during chronic viral infection.

Here we have investigated the role of Treg cell responses in chronic LCMV infection and found that the persistence-prone strain Docile (DOC) induces a substantial dose-dependent expansion of the Treg cell population that directly correlated with the magnitude of the virus inoculum. The expanded Treg cell population massively impaired the functionality of antiviral CD8^+^ T cells, interfered with virus clearance, and thereby promoted chronic viral infection. Strikingly, enforced expansion of the Treg cell population with IL-2/anti-IL-2 immune complexes (IL-2ic; [42]) permitted the persistence of LCMV-DOC already at much lower infection doses and even predisposed to chronic infection with an LCMV-strain that generally fails to establish persistence in immunocompetent mice. Importantly, this infection-triggered expansion of the Treg cell population was greatly inhibited by IL-21R signaling in Treg cells, thus defining a novel role for IL-21 in preventing T cell exhaustion and viral persistence via limiting virus-driven Treg cell proliferation.

## Results

### Dose-dependent expansion of the Treg cell population in response to chronic viral infection

**Figure 1:**
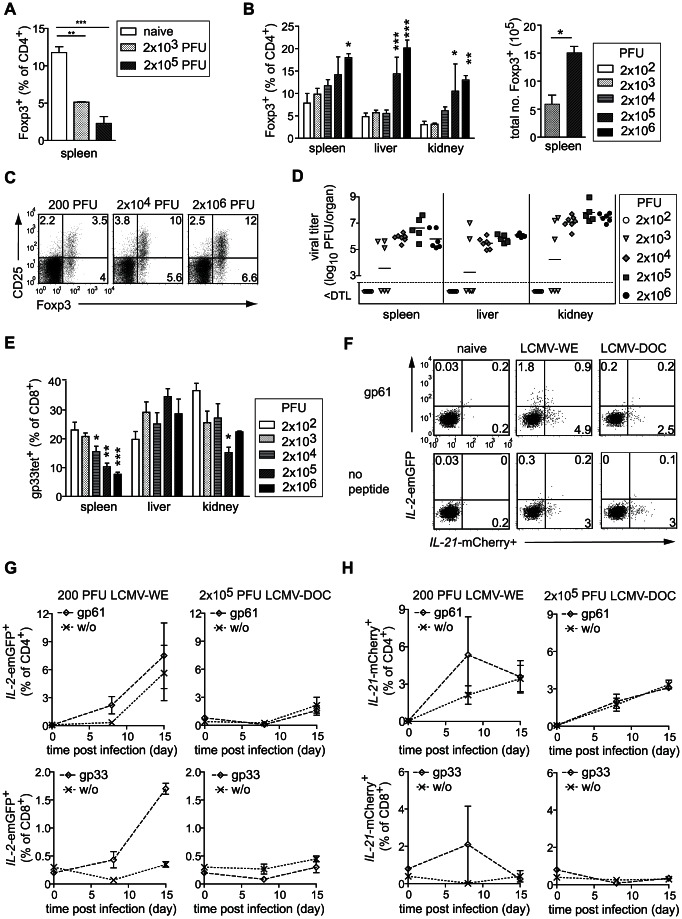
Virus dose-dependent expansion of the Treg cell population in response to chronic viral infection. Characterization of the Foxp3^+^ Treg cell population in spleen (A–C), liver and kidney (B) of C57BL/6 WT mice at days 0 and 7 (A) and at day 15 (B, C) post infection with indicated doses of LCMV-DOC. (A, B) Bar graphs show the mean ±SEM of groups (n = 4–9) of mice. (C) Dot plots gated on CD4^+^ T cells from spleens of individual mice representative for the group. (D) Viral titer in organs of individual mice as determined by plaque forming assay. Dotted horizontal lines indicate the detection limit (DTL). (E) Percentage of gp33-specific CD8^+^ T cells in spleen, liver and kidney at 15 days post infection (dpi). Pooled data from 2–4 independent experiments are shown. Data represent the mean ±SEM of 4–9 mice/group and was compared to mice infected with 200 PFU LCMV-DOC by one-way ANOVA using the Bonferroni correction. (F–H) *Il2*-emGFP-*Il21*-mCherry dual reporter transgenic mice were infected with low dose (i.e. 200 PFU) LCMV-WE or high dose (i.e. 2×10^5^ PFU) LCMV-DOC. (F) Dot plots show IL-2-(emGFP) and IL-21 (mCherry) expression of CD4^+^ T cells in blood of naïve and day 8 LCMV infected mice without or with gp61 restimulation. (G–H) Shown are frequencies of (G) IL-2- or (H) IL-21- expressing CD4^+^ and CD8^+^ T cells without (w/o) or with gp61 or gp33 peptide restimulation at days indicated. Values indicate averages of groups (n = 2–3/group).

We examined the role of Foxp3^+^ Treg cell responses in chronic viral infections in mice infected with the fast-replicating strain of LCMV-DOC. LCMV-DOC is characterized by its potential to establish a chronic, persistent infection depending on the size of the virus inoculum. Although low doses of LCMV-DOC (2×10^2^–2×10^3^ PFU) induce potent CD8^+^ T cell-mediated immunity and are cleared in immunocompetent hosts, infection with intermediate and high doses (2×10^4^–2×10^6^ PFU) results in virus persistence due to the exhaustion of the virus-specific CD8^+^ T cell response [30], [31]. By choosing different virus inocula, we determined dynamics of the Treg cell population during an acute, resolving infection and a chronic infection. After an initial decline of Treg cell numbers between days 0–7 that was independent of the dose of infection and the LCMV strain used (i.e. DOC or WE) (Figure 1A and data not shown), we observed a striking dose-dependent expansion and recovery of the Treg cell population in LCMV-DOC infected mice (Figure 1B, C) that directly correlated with the ability of the virus to establish persistence (Figure 1D). Compared to animals infected with a low dose of LCMV-DOC (200 PFU), mice infected with intermediate (2×10^4^ PFU) or high (2×10^6^ PFU) virus doses exhibited markedly increased proportions of Treg cells in spleen and peripheral organs that amounted up to 20% of all CD4^+^ T cells at 15 days post infection (Figure 1B, C). At this time, infectious virus had been cleared from the blood and organs of all mice infected with 200 PFU and some of the animals infected with 2×10^3^ PFU of LCMV-DOC (Figure 1D and data not shown); while mice infected with persistence-inducing doses (2×10^4^–2×10^6^ PFU) of LCMV-DOC still exhibited high viral titers in blood, spleen, liver and kidney, and subsequently failed to control the infection (Figure 1D and data not shown). As expected, we also detected a dose-dependent reduction in the frequency of gp33-specific CD8^+^ T cells, which was indicative of the progressing exhaustion of the T cell response, particularly in the spleen (Figure 1E), and the resulting inability to resolve the infection (Figure 1D).

Consumption and bioavailability of IL-2 by Treg cells has been suggested to restrict IL-2-dependent effector T cell differentiation and expansion [43], [44], [45], [46]. Conversely, IL-2-driven expansion of CD8^+^ T cell expansion during an immune response can occur at the expense of Treg proliferation/survival [47], [48]. Moreover, IL-21 has been suggested to interfere with Treg-mediated suppression by inhibition of IL-2 [49]. To monitor IL-2 and IL-21 production during acute and chronic infection, we took advantage of *Il2*-emGFP-*Il21*-mCherry dual reporter transgenic mice [50] that were infected with low dose (i.e. 200 PFU) LCMV-WE or high dose (i.e. 2×10^5^ PFU) LCMV-DOC. Both *Il2* (GFP) and *Il21* (mCherry) were predominantly expressed by CD4^+^ compared to CD8^+^ T cells and frequencies increased from days 7–15 post infection with low dose LCMV-WE (Figure 1F–H). Interestingly, chronic infection with high dose LCMV-DOC potently suppressed *Il2*-GFP expression, but did not affect *Il21*-mCherry expression by CD4^+^ T cells (Figure 1F–H).

Together, these data demonstrate a direct correlation between the size of the virus inoculum, CD8^+^ T cell dysfunctionality, virus persistence and the expansion of Treg cells, thus indicating a potential contribution of Treg cells to the impaired T cell function and the induction of viral persistence.

### IL-21 antagonizes the virus-induced Treg cell expansion during chronic infection

**Figure 2:**
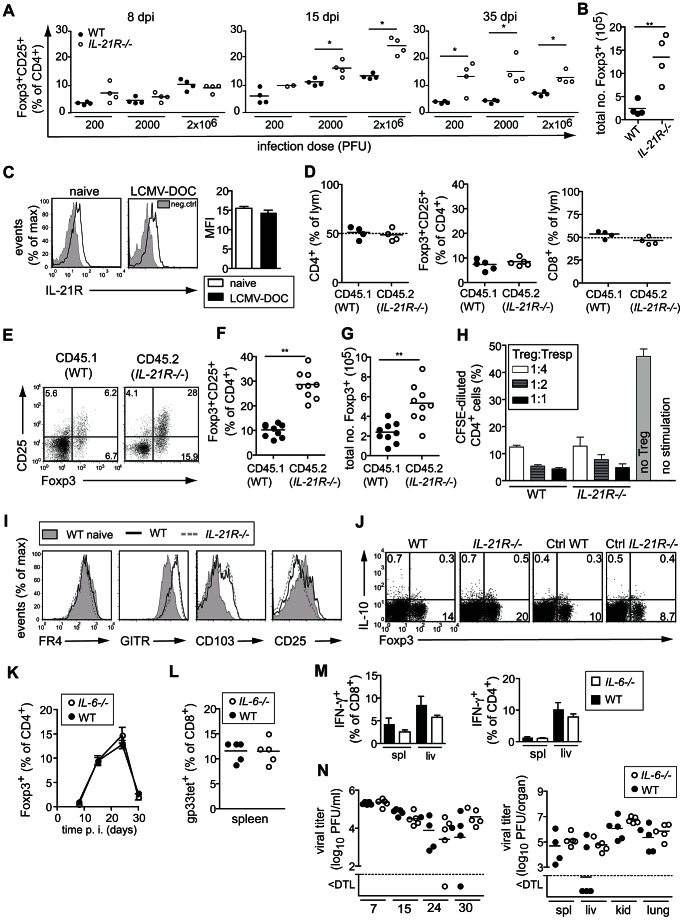
IL-21 antagonizes virus-induced Treg cell expansion during chronic infection. (A) Frequencies of splenic Foxp3^+^CD25^+^CD4^+^ T cells from WT and IL-21R^−/−^ mice at indicated days after infection with indicated doses of LCMV-DOC. (B) Total numbers of Foxp3^+^ cells (2000 PFU LCMV-DOC, 15 dpi). (C) IL-21R expression on GFP^+^CD4^+^Treg cells of naïve and LCMV-DOC infected DEREG mice (2×10^4^ PFU, 15 dpi). The mean fluorescent intensity (MFI) of single mice (left panel) and averages ± SEM of groups (n = 4) (right panel) are shown. (D–G) Analysis of WT (CD45.1^+^):IL-21R^−/−^(CD45.2^+^) mixed BM chimeras. (D) Blood frequencies of CD4^+^, CD8^+^ and Foxp3^+^CD25^+^CD4^+^ T cell populations derived from WT and IL-21R^−/−^ BM 8 weeks after BM transfer prior LCMV infection. Reconstitution efficiency of WT and IL-21R^−/−^ lymphocytes (lym) were in average 51%:49% and values for the CD4^+^ and CD8^+^ T cell populations were corrected for chimerism. (E) FACS plots show CD25 and Foxp3 expression of CD4^+^ T cells, (F) graphs show frequencies and (G) total numbers of splenic Foxp3^+^CD25^+^ Treg cells 35 dpi with 2000 PFU LCMV-DOC. (H) Suppressive activity of FACS-sorted CD4^+^CD25^+^ Treg cells from infected DEREG or DEREGxIL-21R^−/−^ mice was assessed as described in Materials and Methods. (I) Cell surface expression of putative Treg markers and (J) IL-10 production by Foxp3^+^CD4^+^ T cells isolated from WT or IL-21R^−/−^ mice infected with 2×10^4^ PFU LCMV-DOC 15 dpi. (J) Dot plots depict IL-10 production of splenic Foxp3^+^ T cells of WT or IL-21R^−/−^ mice after restimulation with gp61. Shown are representative individuals of groups of mice (n = 4). (K–N) IL-6^−/−^ and WT mice (n = 5/group) were infected with 2×10^4^ PFU LCMV-DOC and analyzed 30 dpi. Shown are frequencies of Foxp3^+^ cells (K), gp33-specific CD8^+^ T cells (L), IFN-γ-producing CD8^+^ and CD4^+^ T cells (M), and virus titers in organs indicated (N). (A–N) Data represent one of two independent experiments.

IL-21 receptor (IL-21R) signaling is essential for the maintenance and sustained functionality of antiviral T cell responses in chronic infections [39], [40], [41]. As a consequence, IL-21R^−/−^ animals have an impaired control of LCMV-DOC, and exhibit much higher virus loads [40]. In LCMV-DOC infected WT mice, the main Treg cell expansion was observed between days 10 to 20 post infection, peaking at 15 days before returning to almost naïve levels by five weeks post infection (Figure 2A, B). Strikingly, this virus-driven Treg cell expansion was much more pronounced and longer lasting in mice lacking IL-21R expression, which suggested that the pro-inflammatory cytokine IL-21 restricted the proliferation of Treg cells in viral infections (Figure 2A, B). Indeed, the Treg cells of naïve and LCMV-DOC infected WT mice expressed the IL-21R as assessed by flow cytometry (Figure 2C). We thus investigated whether the increased Treg expansion observed in IL-21R^−/−^ mice represented a direct inhibitory effect of IL-21 on Treg cells or rather was related to the increased viral replication in these mice. To address this issue, we generated mixed bone marrow (BM) chimeras by reconstituting lethally irradiated WT (CD45.1^+^) mice with a 1∶1 ratio of WT (CD45.1^+^) and IL-21R^−/−^ (CD45.2^+^) BM, and evaluated their Treg cell responses to infection with LCMV-DOC. Analysis of naïve BM chimeras confirmed similar reconstitution efficacy of the CD8^+^ and CD4^+^ T cell populations including Treg cells from both WT and IL-21R^−/−^ donor BMs at 8 weeks after BM transfer (Figure 2D, and data not shown). However, upon infection the population of IL-21R^−/−^ Treg cells expanded 3-fold over that of WT Treg cells to represent 30% versus 10% of all CD4^+^ T cells, respectively (Figure 2E–G). Since this augmented proliferation of IL-21R^−/−^ Treg cells was detected side by side to WT Treg cells in the same animals and identical viral loads, it clearly established the direct inhibitory effect of IL-21 on Treg cells *in vivo*. Nevertheless, we considered that the higher Treg cell numbers in infected IL-21R^−/−^ mice could not only result from the absence of inhibitory IL-21R signaling but might also indicate a compensatory proliferation to overcome a potential functional deficit of the IL-21R^−/−^ Treg cells. To exclude the latter possibility, we isolated CD4^+^CD25^+^ GFP^+^ Treg cells from WT DEREG and IL-21R^−/−^ DEREG mice by FACS-sorting 15 days post infection with 2×10^6^ PFU LCMV-DOC, and compared their suppressive activity in a classical T cell inhibition assay. As shown in Figure 2H, both WT and IL-21R^−/−^ Treg cells comparably inhibited the proliferation of anti-CD3/CD28–stimulated naïve CD25^neg^CD4^+^ T cells *in vitro*, suggesting a normal function of IL-21R^−/−^ Treg cells. Thus, the increased expansion of IL-21R^−/−^ Treg cells in LCMV-DOC infected mice highlighted an important inhibitory role of IL-21 in restraining Treg cell expansion during chronic LCMV infection. Except for a slightly reduced CD25 expression, IL-21R^−/−^ Treg cells were comparable to WT Treg cells with respect to the expression or characteristic Treg cell surface markers (Figure 2I). We did not detect any IL-10 producing or gp61-specific Treg cells in infected mice, suggesting that the suppressive activity of Treg cells in LCMV-DOC infection involved neither IL-10–mediated suppression nor virus-specific Treg cells (Figure 2J and data not shown).

IL-6 has been suggested to regulate the balance between Treg and pro-inflammatory Th17 cell responses [51], [52] similar to IL-21 [53]. While we have previously shown that IL-17–producing CD4^+^ T (Th17) cells were barely detectable in LCMV-DOC infection [40], it remains possible that IL-21 inhibits Treg cell expansion by regulation of IL-6. However, comparing IL-6^−/−^ and WT mice we found no differences in Treg cell expansion (Figure 2K), antiviral CD8^+^ and CD4^+^ T cell responses (Figure 2L, M), and virus titers (Figure 2N) up to day 30 post infection with 2×10^4^ PFU LCMV-DOC. Together with the Treg cell-intrinsic negative IL-21R^−/−^ signaling observed in WT:IL-21R^−/−^ mixed BM chimeras, these data suggest that IL-21 restricted the virus-driven Treg cell expansion in LCMV-DOC infection independently of IL-6 signaling.

### Depletion of Treg cells partially restores CD8^+^ T cell functionality and increases disease severity during chronic viral infection

**Figure 3:**
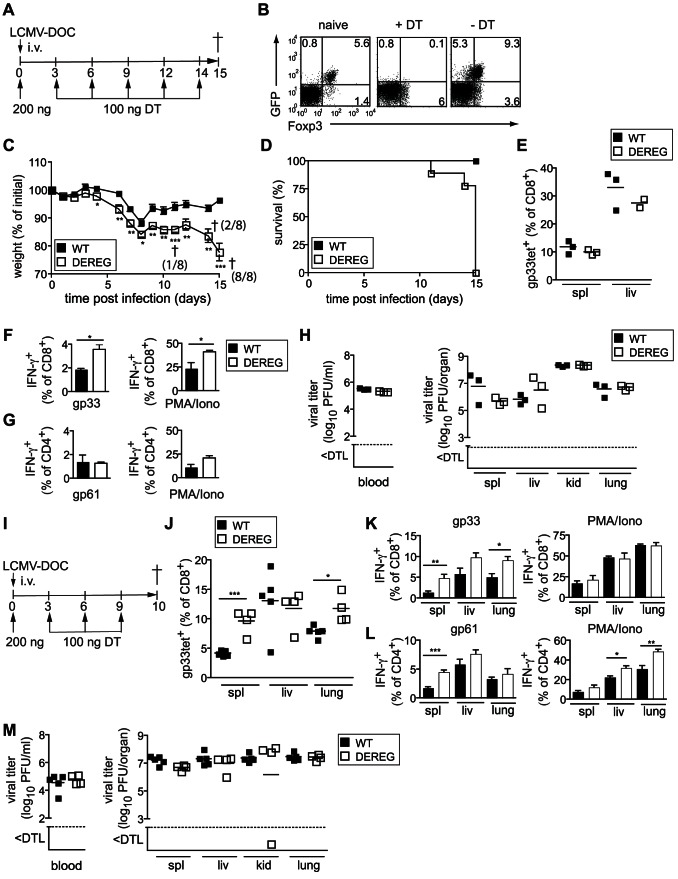
Depletion of Treg cells partially restores CD8^+^ T cell functionality and increases disease severity. DEREG and C57BL/6 WT mice were infected with 2×10^5^ PFU LCMV-DOC and treated with diphtheria toxin (DT) for depletion of Foxp3^+^ cells as illustrated in (A, I). (B) Dot plot of GFP and Foxp3^+^ expressing cells gated on CD4^+^ T cells from naïve and infected mice treated or untreated with DT. (C) Morbidity indicated by weight loss and (D) percentage of surviving mice for groups of 8 mice. (E) Expansion of virus-specific CD8^+^ T cells and cytokine production of splenic (F) CD8^+^ and (G) CD4^+^ T cells as detected with gp33-specific tetramers or intracellular cytokine staining after restimulation with specific peptide or PMA/Ionomycin. (H) Virus titers in blood (left panel) and organs (right panel) of individual mice at 15 dpi. Dotted lines indicate the detection limit (DTL). (J) Percentages of gp33-specific CD8^+^ T cells in spleen, liver, and lungs 10 dpi. Frequencies of splenic IFN-γ-producing CD8^+^ T cells (K) and CD4^+^ T cells (L) after restimulation with specific peptide (left graph) or PMA/Ionomycin (right graph). (M) Virus titers in blood and organs of individual mice 10 dpi. Dotted lines indicate the detection limit (DTL). Data are representative of two independent experiments including n = 8 (A–H) or n = 4 (I–H) mice per group. Symbols depict individual mice and lines indicate averages of groups.

To directly evaluate the impact of Treg cells on viral persistence, we next sought to analyze antiviral T cell responses and viral clearance in the absence of Treg cells. We therefore studied LCMV-DOC infection using the DEREG mouse model, in which Treg cells can be ablated by diphtheria toxin (DT) treatment due to transgenic expression of a high affinity DT receptor under control of the Foxp3 promoter [54]. DEREG and nontransgenic WT control mice were treated with DT and infected with 2×10^5^ PFU LCMV-DOC on day 0, and the DT treatment was continued throughout the experiment (Figure 3A). Since a single DT injection depleted Treg cells in naïve mice for 3 days, we injected DT every 3 days to achieve complete Treg cell ablation. While this treatment effectively depleted all Foxp3^+^ Treg cells in naïve DEREG mice ([54], and data not shown), we consistently observed in LCMV-DOC infected DEREG mice the emergence of a residual GFP^neg^Foxp3^+^ Treg cell population not depleted by DT, presumably due to lacking expression of the GFP-DTR fusion protein (Figure 3B). Compared to DT-treated WT controls, Treg cell-depleted DEREG mice exhibited a greatly enhanced morbidity in response to high dose LCMV-DOC infection, as indicated by an increased weight loss (Figure 3C). As a result, a significant number of DT-treated, LCMV-DOC–infected DEREG mice had to be prematurely removed from the experiment and euthanized, whereas no equivalent morbidity was observed in DT-treated WT mice (Figure 3D). Thus, Treg cell depletion – even if not absolute – substantially aggravated the disease severity in LCMV-DOC–infected DEREG mice. Depletion of Treg cells did not affect the numbers of virus-specific CD8^+^ T cells, as indicated by the percentage of gp33-specific CD8^+^ T cells in spleens and livers (Figure 3E). However, Treg cell-depletion to some extent restored the functionality of the antiviral CTL and significantly increased the frequencies of gp33-specific and overall splenic CD8^+^ T cells producing IFN-γ upon restimulation *in vitro* (Figure 3F). In comparison, Treg cell-depletion did not enhance frequencies of IFN-γ–producing virus-specific CD4^+^ T cells (Figure 3G). In spite of partly restoring CD8^+^ T cell cytokine responses, Treg cell depletion did not influence virus control, and LCMV-DOC replicated to comparably high levels in the blood and organs of DT-treated WT and DEREG mice at 15 days post infection (Figure 3H). Thus, Treg cells appeared to inhibit the functionality rather than the expansion of antiviral T cells. Consistent with the observed onset of immunopathology, the effect of Treg cell depletion on the antiviral T cell response was more pronounced at 10 days post infection (Figure 3I–M). Depletion of Treg cells resulted in higher percentages of gp33-specific CD8^+^ T cells (Figure 3J) and increased percentages of cytokine-producing antiviral CD8^+^ and CD4^+^ T cells (Figure 3K, L), yet did not affect viral titers (Figure 3M).

**Figure 4:**
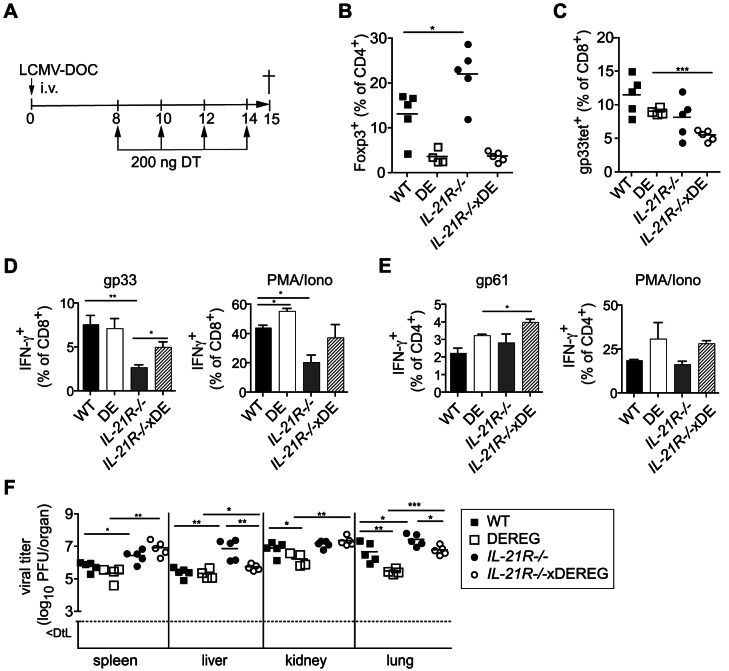
Depletion of Treg cells partially restores CD8^+^ T cell functionality in WT and IL-21R^−/−^ mice. DEREG, C57BL/6 (WT), DEREGxIL-21R^−/−^ and IL-21R^−/−^ mice were infected with 2000 PFU LCMV-DOC and treated with diphtheria toxin (DT) at indicated days before analysis at day 15 as illustrated in (A). (B) Percentages of Foxp3^+^ Treg cells and (C) gp33-specific CD8^+^ T cells in the spleen. (D, E) Frequency of IFN-γ-producing CD8^+^ (D) and CD4^+^ (E) T cells as assessed by intracellular cytokine staining after *in vitro* restimulation with specific peptide or PMA/Ionomycin. (F) Shown are virus titers in organs of individual mice 15 dpi. Dotted lines indicate the detection limit (DTL). Dots represent individual mice, the lines the averages (*p≤0.05).

We next assessed the impact of increased Treg cell numbers in IL-21R^−/−^ mice and treated both IL-21R^−/−^ and WT mice with DT between days 8 to 15 post infection with 2000 PFU LCMV-DOC (Fig. 4A). Although Treg cells were considerably (but not entirely) depleted, frequencies of gp33-specific CD8^+^ T cells remained unchanged (Figure 4B, C) similar to the results obtained by depletion through the entire course of infection (Figure 3B, E). However, Treg cell depletion partially restored frequencies of IFN-γ-producing gp33-specific cells in IL-21R^−/−^ mice to levels found in WT mice (Figure 4D). Furthermore, Treg cell depletion lowered virus titers significantly in liver and lung of IL-21R^−/−^ mice, although viral loads in spleen and kidney remained unaffected (Figure 4F).

Whether the differences in antiviral CD8^+^ T cells were too small to result in better virus control or whether the failure to detect differences in viral titers (Figure 3H, M) has to be attributed to the suppressive activity of the residual Treg cells that resisted DT depletion (Figures 3B and 4B) remains to be clarified. Regardless, our results establish a link between the functional impairment of the CD8^+^ T cell response and the elevated Treg cell levels observed during chronic infection in absence of IL-21.

### IL-2 driven Treg cell expansion inhibits antiviral T cell responses and promotes persistent viral infection

**Figure 5:**
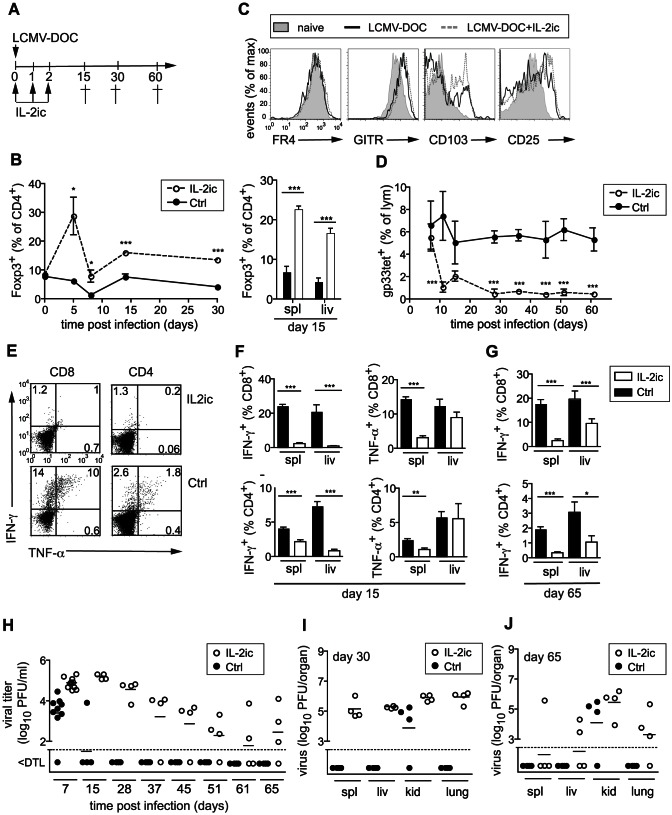
Forced Treg cell expansion inhibits antiviral T cell responses and interferes with effective virus clearance. (A) Mice were infected with 2000 PFU LCMV-DOC and treated with IL-2/anti-IL-2mAb immune complexes (IL-2ic) or untreated (ctrl) as illustrated. (B) Frequencies of Treg cells measured longitudinally in blood (left) and at 15 dpi in spleen and liver (right). (C) Cell surface expression of putative Treg markers on Foxp3^+^CD4^+^ T cells of individuals out of a group (n = 4) of mice. (D) Percentages of gp33-specific CD8^+^ T cells in blood at days indicated. (E–G) Frequency of IFN-γ- and TNF-α-producing CD8^+^ and CD4^+^ T cells after *in vitro* restimulation with gp33 and gp61 peptides at day 15 (E–F) and day 65 (G) post infection (H–J). Values show virus titers in blood at days indicated (H) and in organs of individual mice at days 30 (I) and 65 (J) post infection. Dotted lines indicate the detection limit (DTL). Data are representative of two (A–B) or three (C–I) independent experiments. Symbols represent individual mice and lines averages of the groups.

We next examined the potential of Treg cells to limit the antiviral immune response and to promote virus persistence in a gain of function approach. For this purpose, we injected immune complexes (ic) comprised of recombinant IL-2 and the anti-IL-2 antibody JES6-1 [42], to selectively expand the subset of Foxp3^+^CD4^+^ Treg cells *in vivo* (Figure 5A). In naïve mice, three injections of IL-2ic drastically expanded the Treg population to represent 40–50% of all CD4^+^ T cells in blood, spleen and liver within 5 days after the first injection (Figure S1 and Figure 5B). Similarly, IL-2ic treatment triggered a pronounced expansion of Treg cells in mice infected with 2×10^3^ PFU LCMV-DOC, established stably elevated levels of Treg cells in blood for at least 30 days (Figure 5B), and thus appeared comparable to that observed during high dose LCMV-DOC infection. The Treg cell population of infected, IL-2ic-treated mice was fully comparable to that of untreated, infected mice, with respect to cell surface expression of FR4, GITR, CD103 and CD25 (Figure 5C) as well as TCR–Vβ profiles (data not shown). The IL-2ic-stimulated expansion of the Foxp3^+^ Treg cell population profoundly interfered with generation and maintenance of gp33-specific CD8^+^ T cells, CD62L downregulation, and their capacity to produce IFN-γ and TNF-α as measured in spleen and liver at days 15, 30, and 65 post infection (Figure 5D–G, and Figure S2), which is reminiscent of the state of exhaustion that usually coincides with viral persistence in high dose LCMV-DOC infection. Accordingly, this long lasting impairment of antiviral T cells in presence of the enhanced Treg cell expansion prevented IL-2ic–treated animals from controlling a low dose LCMV-DOC infection. While infectious virus was readily cleared from the blood and most organs in control mice within 15 days, IL-2ic-induced Treg cell expansion resulted in a failure to clear virus in spleens, livers, kidneys and lungs for more than 2 months (Figure 5H–J). Notably, IL-2ic expanded Treg cells also impaired antiviral CD8^+^ T cell effector responses and viral clearance of low dose LCMV-WE infection, which is otherwise rapidly cleared irrespectively of the viral inoculate (Figure S3).

Taken together, the experimental expansion of Treg cells recapitulated both the long lasting functional impairment of the antiviral T cell response and the viral persistence that characterize high-dose LCMV-DOC infection in the setting of a low dose inoculum, and thus emphasized the remarkable potency of Treg cells to facilitate persistent viral infections. While Sprent and colleagues have clearly shown that IL-2:IL-2mAb (JES6-1) primarily target high affinity IL-2R^+^ Treg cells and have minimal effects on low affinity IL-2R^+^ naïve and memory CD8^+^ T cells [42], we cannot completely rule out the possibility that the IL-2:IL-2mAb also target high affinity antiviral effector CD8^+^ T cells resulting in terminal differentiation and exhaustion. However, this scenario appears unlikely considering the time of treatment with the IL-2:IL-2mAb complexes (i.e. days 0–2), their short half-life (i.e. 4 h) [42], and the normal expansion of antiviral CD8^+^ T cells until day 7.

**Figure 6:**
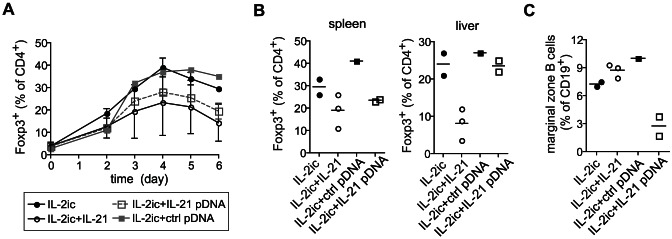
IL-21 delivery impairs IL-2ic driven expansion of Treg cells. Expression vectors encoding the IL-21 gene fused with the hIgG1 (IL-21pDNA) or a control hIgG1 gene (ctrl pDNA) alone were delivered to C57BL/6 mice by hydrodynamic injection 24 h prior injection of IL-2ic. Alternatively, IL-2ic treated C57BL/6 WT mice were injected with 2 µg of IL-21-hIgG1 fusion protein consecutively every 12 h from days 0–5. Foxp3^+^ Treg cells were measured (A) in the blood at days indicated and (B) in spleen and liver at day 6 by flow cytometry. (C) Marginal zone B cells in the spleen at day 6. Values show percentages of Foxp3^+^ T cells of CD4^+^ T cells (A, B) and CD21^+^CD23^low^ of CD19^+^ cells (C). Dots represent individual mice and lines indicate averages (*p≤0.05).

The above data demonstrate the differential regulation of the Treg cell population during viral infection by IL-2 and IL-21. To better understand counter-regulation of Treg cells by IL-2 and IL-21 in the absence of confounding virus dynamics, we delivered the IL-21 gene by hydrodynamic injection to IL-2ic treated mice in the absence of viral infection (Figure 6). Indeed, IL-21 significantly inhibited IL-2ic driven expansion of Treg cells. Similar results were obtained by co-injection of an engineered IL-21-Fc fusion protein together with IL-2ic. Together, these data indicate that IL-21 interferes with IL-2 driven expansion of Treg cells to optimize antiviral effector T cell responses.

## Discussion

T cell exhaustion represents a state of T cell dysfunction associated with clinically relevant diseases, such as persistent viral infections or cancer. Even though the molecular signature of exhausted T cells has been characterized in detail at the functional and transcriptional level [32], [34], we are only beginning to understand the immunological mechanisms that support or counteract the development of T cell exhaustion during chronic infections [35]. In this study we report two major findings that establish a pathway of T cell exhaustion mediated by Treg cells during viral infection, and indicate its modulation by both, the pathogen and the host. First, we show that a persistence-inducing virus triggers the massive proliferation of Treg cells between days 9–20 post infection and demonstrate the potential of Treg cells to promote T cell exhaustion and chronic infection. Second, we identify IL-21 as a crucial host factor that antagonizes this virus-driven expansion of the Treg cell population. Together, these results suggest enhanced Treg cell responses as a mechanism of immune evasion that could be therapeutically targeted with IL-21.

Treg cells are essential for immune homeostasis and T cell tolerance [8], [9], yet their contribution to anti-infectious immune responses is poorly defined. In the setting of persistent viral infections that was investigated here, Treg cells appear to down-regulate antiviral T cell responses, and thus prevent the potentially lethal immunopathology caused by prolonged immune activation in presence of highly replicating virus (Figure 3). Supporting this notion, clinical studies have associated high numbers of Treg cells to chronic infection with HIV [29], HCV [28], [55], or HBV [56], whereas low Treg cell numbers have been reported for elite HIV controllers [57], which collectively suggests that Treg cells modulate the equilibrium between host immune response and persisting virus [27] [34]. Our study has now recapitulated these observations in a murine model of persistent viral infection and applied experimental manipulation of the Treg cell response to define the direct link between virus-induced Treg cell expansion and T cell exhaustion. Our initial observation that titrated doses of LCMV-DOC induce graded degrees of Treg cell proliferation and T cell exhaustion allowed us to assess the impact of such virus-induced Treg cell expansion under “non-persisting conditions” using the identical LCMV strain. Although the expansion of Treg cells with IL-2ic achieved very high Treg cell numbers, it is important to note that this level of Treg cell expansion was fully comparable to that induced with LCMV-DOC at high doses or in IL-21R^−/−^ mice. The Treg cell responses triggered by LCMV-DOC in presence or absence of IL-2ic treatment did not phenotypically differ with respect to their cell surface marker expression and exhibited TCRβ profiles similar to that described for LCMV clone 13 infection [58]. Thus, the IL-2ic-elicited Treg cell proliferation appeared to truly mimic the physiological Treg cell expansion during chronic LCMV-DOC infection.

These experiments exposed two facets of the antiviral Treg cell response in LCMV-DOC infected mice that are central for our understanding of the role and potential of Treg cells within antiviral and anti-tumoral responses. First, the increased morbidity in Treg-depleted DEREG mice infected with LCMV-DOC clearly revealed the critical role of Treg cells for preventing lethal immunopathology caused by potent immune responses to persisting antigen. Second, both the depletion as well as the gain of function approach demonstrated that Treg cells primarily modulate the functionality (e.g. cytokine response, antiviral activity) of antiviral T cells rather than influencing their priming consistent with an earlier report showing compromised cytolysis but no defect in priming, proliferation and motility of regulated CTLs [59]. This finding is especially promising, since it implies that the primed but exhausted antiviral T cells present in chronically infected subjects could be therapeutically rescued by removal of Treg cell-mediated suppression.

Dynamics of regulatory and effector T cell populations in homeostasis and during an immune response are very sensitive to the availability of IL-2. Competition for IL-2 between effector and regulatory T cells has been suggested to control tolerance and immunity or the outcome of infectious disease. Associated with the rapid expansion of virus-specific CD8^+^ T cells in the early phase of infection between days 0 to 8, we observed a remarkable drop in Treg cells below naïve levels irrespective of the LCMV inocula and strain used. This has also been observed in other infections and suggested to be due to consumption of IL-2 by expanding CD8^+^ T cells and required for efficient clearance of the invader [47], [48]. While this appears feasible, it should be noted that expansion of CD8^+^ T cells in the acute phase of LCMV infection is independent of IL-2 [60], [61] and that hyper-proliferation of Treg cells between days 10–20 in chronic LCMV infection was associated with potent suppression of IL-2 production by CD8^+^ T cells (Figure 1F, G) arguing that IL-2 availability does not sufficiently explain cross-regulation of effector CD8^+^ T cells and Treg cell proliferation in acute and chronic LCMV infection. *In vitro* experiments suggested that IL-21 could inhibit Treg cells by suppression of IL-2 production in CD4^+^ effector T cells [49]. However, our results in the WT:IL-21R^−/−^ mixed BM chimeric mice demonstrate that IL-21 inhibits Treg cell expansion directly in a cell intrinsic manner (Figure 2E–G). The finding that expansion of Treg cells induced by IL-2ic treatment was impaired by simultaneous (hydrodynamic) overexpression of the IL-21 gene (Figure 6) further supports this conclusion. Thus, IL-2 and IL-21 exert opposing activities on Treg cells, while they cooperate in driving effector and memory T cell responses, which adds another level of complexity to theoretical and experimental models addressing the dynamics of Treg cells and effector T cells [45].

Amongst the known Treg cell effector molecules, IL-10 has been shown to support the functional impairment of T cell responses during chronic infection with LCMV clone 13 [36], [37]. However, we were unable to detect any IL-10-producing Treg cells in LCMV-DOC infected mice (Figure 2J). Furthermore, IL-10 blocking antibodies or genetic IL-10-deficiency did not prevent T cell exhaustion and viral clearance in response to LCMV-DOC, in contrast to LCMV clone 13 infection [62]. The exhausted T cells in chronically LCMV infected mice have been shown to upregulate expression of several co-inhibitory receptors, e.g. PD-1 and Tim3, which contribute to T cell exhaustion in the LCMV model [63], [64] and in human HIV patients [65], [66]. It will thus be important to test whether these pathways are involved in the Treg cell-induced T cell exhaustion described in our study. Though a detailed characterization is beyond the scope of the current analysis, the experiments described in this manuscript will provide the framework for further mechanistic studies.

IL-6 and IL-21 have similar activities and interact in the cross-regulation of inducible Treg and Th17 cell development *in vitro* and *in vivo* depending on the experimental model [51], [52], [53], [67], [68]. Interestingly, while IL-6 has recently also been shown to be essential for viral control by enhancing follicular T helper cell responses at late stages of chronic infection [69], virus titers and Treg numbers were comparable in LCMV-DOC infected IL-6^−/−^ and WT mice up to day 30 post infection (Figure 2K–N) [69]. Therefore, during chronic LCMV-DOC infection, the inhibition of virus-induced Treg cell expansion is a distinct function of IL-21, which is not accompanied by elevated IL-17 production of CD4^+^ T cells [40].

The importance of cell-intrinsic IL-21R signaling for the maintenance of CD8^+^ T cell functionality has been well documented [39], [40], [41] and is considered as the primary effect of IL-21 promoting the immune control of chronic viral infections. However, the present report clearly demonstrates that besides this direct function on CD8^+^ T cells, IL-21 also efficiently restricts the virus-induced expansion of the Treg population in a cell intrinsic manner. It remains to be clarified to which extent the direct effects of IL-21 on CD8^+^ T cells and its indirect effects on CD8^+^ T cells via the inhibition of Treg cells differentially contribute to the overall protective function of IL-21 in chronic viral infections considering that we only achieved limited recovery of T cell functionality (e.g. regain of cytokine production) and improved viral clearance only in lung and liver but not spleen and kidney of IL-21R^−/−^ mice by Treg depletion. It should be noted, however, that, for reasons unknown, a fraction of (GFP^neg^Foxp3^+^) Treg cells resisted depletion by diphtheria toxin during LCMV infection in both IL-21R^−/−^ and WT mice, although Treg depletion in naive mice was almost complete (>98%). It remains to be investigated whether the undeletable GFP^neg^Foxp3^+^ cells can compensate for the deleted Treg cells or represent a subpopulation of Treg cells that is responsible for maintenance of regulatory activity in LCMV-infected DEREG mice.

Regardless, our findings suggest a dual importance of IL-21 for preventing T cell exhaustion during chronic viral infections, and demonstrate that IL-21 in addition to its known direct effects on antiviral T cells [39], [40], [41] also partially alleviates the suppressive activity of Treg cells. Notably, in a model of acute lung infection, it was recently demonstrated that IL-21R^−/−^ mice are protected from fatal lung immunopathology induced by pneumonia virus [70]. It is tempting to speculate that IL-21 might aggravate immunopathology by suppression of Treg cells in this infection model.

In summary, our data support the concept of virus-induced Treg cell expansion as an active immune evasion strategy, and thus highlight a novel pathway by which viruses exploit regulatory mechanisms of the immune system to establish persistent infection.

In view of the relevance to human disease these results have direct therapeutic implications and suggest strategies that boost IL-21 signaling in T cells as novel treatment options for chronic viral infections and cancer.

## Materials and Methods

### Viruses and mice

The LCMV strains WE and DOC were originally provided by Rolf Zinkernagel (University of Zurich, Switzerland) and were propagated on L929 or MDCK cells, respectively. C57BL/6 WT and IL-6^−/−^ mice [71] were from Charles River Inc. SMARTA-2 mice (expressing a transgenic TCR specific for LCMV-GP 61–80; [72] and IL-21R^−/−^ mice [73] were bred locally. DEREG-mice [54] were kindly provided by Tim Sparwasser (TWINCORE, Hannover, Germany) and crossed with the IL-21R^−/−^ strain at our facility. *Il21*-mCherry/*Il2*-emGFP dual-reporter transgenic mice [50] were kindly provided by Warren Leonard, National Institutes of Health, Bethesda, MD, USA. Mice were housed in individually ventilated cages under specific pathogen free conditions at BioSupport AG (Zurich, Switzerland). For the generation of BM chimeras, recipient mice were lethally irradiated (9.5 Gy, using a cesium source) one day before reconstitution with 1×10^7^ CD4/CD8-depleted (Miltenyi Biotec) BM cells. Mice were infected i.v. with the indicated virus doses.

Ethics statement: All animal experiments were approved by the local animal ethics committee (Kantonales Veterinärsamt Zürich, licenses 217/2008 and 113/2012), and performed according to local guidelines (TschV, Zurich) and the Swiss animal protection law (TschG).

### Cells and reagents

All cell lines were originally obtained from the American Tissue Culture Collection (ATCC). Chemicals were purchased from Sigma-Aldrich except were otherwise stated. PE- and APC-conjugated peptide-MHC class I tetramers (H-2D^b^/gp33-41) were generated as described [74] or kindly provided the NIH tetramer core facility. The LCMV-GP peptides gp33-41 (KAVYNFATM) and gp61-80 (GLNGPDIYKGVYQFKSVEFD) were bought from Mimotopes. The following antibodies (all eBioscience unless otherwise stated; clone names given in parentheses) were used for flow cytometry: FITC-labeled anti-CD4 (L3T4), anti-CD62L (MEL-14); PE-labeled anti-CD4 (GK1.5; conjugated in our laboratory), anti-CD8α (53-6.7; BioLegend), anti-CD25 (PC61), anti-GITR (DTA-1; BioLegend), anti-CD103 (2E7), anti-FR4 (12A5; BioLegend), anti-IL21R-biotin (4A9) – Streptavidin-RPE (BioLegend) and anti-TNF-α (MP6-XT22); PerCP-labeled anti-CD4 (RM4-5; BioLegend), anti-CD45.1 (A20; BioLegend), anti-CD8 (53-6.7; BD); APC-labeled anti-CD4 (GK1.5), anti-CD127 (SB/199; Biolegend), anti-CD8 (53-6.7; BioLegend), anti-CD45.2 (104), anti-IL21R (4A9; BioLegend), anti-Foxp3 (FJK-16S), anti-IFN-γ (XMG1.2; BioLegend), anti-IL-2 (JES6-5H4), anti-IL-10 (JES5-16E3).

### Manipulation of Treg cells

In depletion experiments, DEREG and WT control mice were i.p. injected with DT (Merck) diluted in PBS. After an initial dose of 200 ng DT, mice were treated with 100 ng DT every third day unless otherwise indicated. To boost Treg cells, mice received 3 daily i.p. injections of IL-2ic generated from carrier-free recombinant mouse IL-2 and anti-IL-2 mAb (JES6-1A12; both from eBioscience) as described [42].

### Flow cytometry

Tetramer and antibody staining was performed on blood cells and single cell suspensions prepared from organs. Spleens and kidneys were passed through a 70 µM cell strainer to obtain single cell suspensions. Livers were first dissected into small pieces, and then passed through a cell strainer before lymphocytes were purified by Lympholyte M gradient centrifugation (Cedarlane Laboratories Ltd.). Blood samples were pretreated with red blood lysis buffer (155 mM NH_4_Cl, 10 mM KHCO_3_, 0.1 mM EDTA, pH 7) for 10 min at RT. Cells were incubated with anti-CD16/CD32 mAb (2.4G2) to block FcγR. For surface staining, cells were incubated at RT with peptide MHC I tetramers in FACS buffer (FB; PBS containing 0.5% BSA) for 15 min followed by addition of the relevant surface antibodies and incubation for additional 20 min at 4°C. Cytokine-production by T cells was assessed using intracellular cytokine staining of single cell suspensions that had been stimulated in presence of 2 µg/ml Monensin with either 1 µM specific peptide or 100 ng/ml PMA and 1 µg/ml Ionomycin for 4 hours *in vitro*. The cells were surface-stained, fixed with 4% Formaldehyde in PBS and permeabilized with permeabilization buffer (FB containing 1% Saponin). Intracellular staining was then performed in permeabilization buffer at 4°C for 20 minutes. After 2 washes with permeabilization buffer, cells were resuspended in FB. All samples were acquired on a FACSCalibur with CellQuest software (both BD Biosciences) and analyzed using the FlowJo software (Tree Star Inc.).

### Determination of virus titers

Blood samples were obtained from LCMV-infected mice at indicated times, diluted 5-fold in MEM (5% FCS) containing 50 U.I. of Liquemin (Drossapharm) and frozen. Organs were collected in 1 ml MEM (5% FCS) and smashed with a Tissue Lyser (Qiagen). Samples were stored at −80°C until further analysis by plaque forming assay [75].

### Treg cell suppression assay

Responder CD4^+^ T cells were purified from naïve spleens by positive MACS separation (Miltenyi Biotec) and labeled with 25 µM CFSE (Molecular Probes, C-1157) at a density of 10^6^ cells/ml in PBS containing 0.5% BSA for 7 min at RT. The labeling reaction was stopped with pure FCS and cells were washed twice with IMDM containing 10% FCS. As suppressor cells, CD25^+^ Treg cells were FACS-sorted from MACS-purified CD4^+^ T cells isolated from LCMV-DOC infected DEREG and IL-21R^−/−^ DEREG mice. CFSE-labeled responder CD4^+^ T cells (1×10^5^/well) and sorted Treg cells were then incubated at defined responder/suppressor ratios (1∶1, 2∶1, 4∶1) in RPMI (10% FCS, 50 µM β-ME and 100 U/ml IL-2) for 6 days in the presence of 5×10^6^ anti-CD3/CD28-coated (both eBioscience) latex beads.

### Hydrodynamic gene delivery

Mouse IL-21 coding sequence was amplified by PCR and linked with hIgG1 Fc domain and cloned into pLIVE *in vivo* expression vector (Mirus Bio). Endotoxin-free plasmid DNA (100 µg) was injected i.v. in PBS in a volume equal to 10% body weight (0.1 ml/g) within 5 s. As a control, a hIgG1 expression vector was injected. To supplement IL21, mice received i.p. injections of 2 µg recombinant IL-21-hIgG1 fusion protein (kindly provided by Daniel Christ, Garvan Institute for Medical Research, Sydney, Australia) or PBS two times daily.

### Statistical analysis

Data are shown as average ±SEM. Statistical analysis was performed with the unpaired two-tailed *t*–test (except for Fig. 1B–E) using the Prism 4.0 software (GraphPad Software). Differences were considered significant for *p*<0.05 and were denoted as *, *p*<0.05; **, *p*<0.01; ***, *p*<0.001.

## Supporting Information

Figure S1 **Expansion of Treg cells with IL-2ic.** Mice were treated with IL-2ic as depicted in (A). (B) Treg cell populations in blood, spleens, and livers of treated as compared to untreated (ctrl) mice. Shown are dot plots of representative individuals of groups (n = 4). Percentages and total cell counts of Foxp3 cells are indicated in quadrants.
(EPS)

Figure S2 **Expanded Treg cell population impairs the antiviral T cell response and promotes virus persistence.** (A–C) Kinetics of CD8^+^ and CD4^+^ T cells during infection with 2000 PFU LCMV-DOC in presence or absence of IL-2ic-mediated Treg cell expansion. Shown are percentages and activation status of (A) CD8^+^ T cells, (B) gp33-specific CD8^+^ T cells and (C) Foxp3-negative CD4^+^ T cells in blood. Lym, lymphocytes. Lines indicate averages of groups. Data are representative of two independent experiments (n = 4/group).
(EPS)

Figure S3 **Treg cells promote virus persistence in a model of acute viral infection.** (A) Percentages of gp33-specific CD8^+^ T cells in blood, spleens and livers of mice infected with 200 PFU LCMV-WE in the presence or absence of IL-2ic-mediated Treg cell expansion. (B, C) Percentage of IFN-γ- and TNF-α-producing virus-specific CD8^+^ and CD4^+^ T cells at (B) 15 and (C) 29 dpi as assessed by intracellular cytokine staining after restimulation with gp33 or gp61 peptide, respectively. Dot plots show representative mice and bar graphs indicate means ±SEM of groups (n = 3–4) of mice. (D) Virus titers in blood and organs of individual mice as determined by plaque forming assay 15 dpi. Dotted lines indicate the detection limit (DTL). Data are representative of two independent experiments.
(EPS)

## References

[1] Sakaguchi S, Sakaguchi N, Asano M, Itoh M, Toda M (1995) Immunologic self-tolerance maintained by activated T cells expressing IL-2 receptor alpha-chains (CD25). Breakdown of a single mechanism of self-tolerance causes various autoimmune diseases. J Immunol 155: 1151–1164. 7636184

[2] Fontenot JD, Gavin MA, Rudensky AY (2003) Foxp3 programs the development and function of CD4+CD25+ regulatory T cells. Nat Immunol 4: 330–336. 12612578 10.1038/ni904

[3] Hori S, Takahashi T, Sakaguchi S (2003) Control of autoimmunity by naturally arising regulatory CD4+ T cells. Adv Immunol 81: 331–371. 14711059 10.1016/s0065-2776(03)81008-8

[4] Khattri R, Cox T, Yasayko SA, Ramsdell F (2003) An essential role for Scurfin in CD4+CD25+ T regulatory cells. Nat Immunol 4: 337–342. 12612581 10.1038/ni909

[5] Sakaguchi S, Sakaguchi N, Asano M, Itoh M, Toda M (1995) Immunologic self-tolerance maintained by activated T cells expressing IL-2 receptor alpha-chains (CD25). Breakdown of a single mechanism of self-tolerance causes various autoimmune diseases. J Immunol 155: 1151–1164. 7636184

[6] Hori S, Nomura T, Sakaguchi S (2003) Control of regulatory T cell development by the transcription factor Foxp3. Science 299: 1057–1061. 12522256 10.1126/science.1079490

[7] Fontenot JD, Rasmussen JP, Williams LM, Dooley JL, Farr AG, et al (2005) Regulatory T cell lineage specification by the forkhead transcription factor foxp3. Immunity 22: 329–341. 15780990 10.1016/j.immuni.2005.01.016

[8] Rudensky AY (2011) Regulatory T cells and Foxp3. Immunol Rev 241: 260–268. 21488902 10.1111/j.1600-065X.2011.01018.x PMC3077798

[9] Sakaguchi S, Yamaguchi T, Nomura T, Ono M (2008) Regulatory T cells and immune tolerance. Cell 133: 775–787. 18510923 10.1016/j.cell.2008.05.009

[10] Tang Q, Bluestone JA (2008) The Foxp3+ regulatory T cell: a jack of all trades, master of regulation. Nat Immunol 9: 239–244. 18285775 10.1038/ni1572 PMC3075612

[11] Bennett CL, Christie J, Ramsdell F, Brunkow ME, Ferguson PJ, et al (2001) The immune dysregulation, polyendocrinopathy, enteropathy, X-linked syndrome (IPEX) is caused by mutations of FOXP3. Nat Genet 27: 20–21. 11137993 10.1038/83713

[12] Brunkow ME, Jeffery EW, Hjerrild KA, Paeper B, Clark LB, et al (2001) Disruption of a new forkhead/winged-helix protein, scurfin, results in the fatal lymphoproliferative disorder of the scurfy mouse. Nat Genet 27: 68–73. 11138001 10.1038/83784

[13] Wildin RS, Ramsdell F, Peake J, Faravelli F, Casanova JL, et al (2001) X-linked neonatal diabetes mellitus, enteropathy and endocrinopathy syndrome is the human equivalent of mouse scurfy. Nat Genet 27: 18–20. 11137992 10.1038/83707

[14] Freyschmidt EJ, Mathias CB, Diaz N, MacArthur DH, Laouar A, et al (2010) Skin inflammation arising from cutaneous regulatory T cell deficiency leads to impaired viral immune responses. J Immunol 185: 1295–1302. 20548030 10.4049/jimmunol.0903144 PMC3873154

[15] Fulton RB, Meyerholz DK, Varga SM (2010) Foxp3+ CD4 regulatory T cells limit pulmonary immunopathology by modulating the CD8 T cell response during respiratory syncytial virus infection. J Immunol 185: 2382–2392. 20639494 10.4049/jimmunol.1000423 PMC2923480

[16] Lanteri MC, O'Brien KM, Purtha WE, Cameron MJ, Lund JM, et al (2009) Tregs control the development of symptomatic West Nile virus infection in humans and mice. J Clin Invest 119: 3266–3277. 19855131 10.1172/JCI39387 PMC2769173

[17] Suvas S, Azkur AK, Kim BS, Kumaraguru U, Rouse BT (2004) CD4+CD25+ regulatory T cells control the severity of viral immunoinflammatory lesions. J Immunol 172: 4123–4132. 15034024 10.4049/jimmunol.172.7.4123

[18] Dietze KK, Zelinskyy G, Gibbert K, Schimmer S, Francois S, et al (2011) Transient depletion of regulatory T cells in transgenic mice reactivates virus-specific CD8+ T cells and reduces chronic retroviral set points. Proc Natl Acad Sci U S A 108: 2420–2425. 21262821 10.1073/pnas.1015148108 PMC3038736

[19] Shafiani S, Tucker-Heard G, Kariyone A, Takatsu K, Urdahl KB (2010) Pathogen-specific regulatory T cells delay the arrival of effector T cells in the lung during early tuberculosis. J Exp Med 207: 1409–1420. 20547826 10.1084/jem.20091885 PMC2901066

[20] Suffia IJ, Reckling SK, Piccirillo CA, Goldszmid RS, Belkaid Y (2006) Infected site-restricted Foxp3+ natural regulatory T cells are specific for microbial antigens. J Exp Med 203: 777–788. 16533885 10.1084/jem.20052056 PMC2118233

[21] Suvas S, Kumaraguru U, Pack CD, Lee S, Rouse BT (2003) CD4+CD25+ T cells regulate virus-specific primary and memory CD8+ T cell responses. J Exp Med 198: 889–901. 12975455 10.1084/jem.20030171 PMC2194203

[22] Zelinskyy G, Dietze KK, Husecken YP, Schimmer S, Nair S, et al (2009) The regulatory T-cell response during acute retroviral infection is locally defined and controls the magnitude and duration of the virus-specific cytotoxic T-cell response. Blood 114: 3199–3207. 19671923 10.1182/blood-2009-03-208736

[23] Lund JM, Hsing L, Pham TT, Rudensky AY (2008) Coordination of early protective immunity to viral infection by regulatory T cells. Science 320: 1220–1224. 18436744 10.1126/science.1155209 PMC2519146

[24] Dittmer U, He H, Messer RJ, Schimmer S, Olbrich AR, et al (2004) Functional impairment of CD8(+) T cells by regulatory T cells during persistent retroviral infection. Immunity 20: 293–303. 15030773 10.1016/s1074-7613(04)00054-8

[25] Manigold T, Shin EC, Mizukoshi E, Mihalik K, Murthy KK, et al (2006) Foxp3+CD4+CD25+ T cells control virus-specific memory T cells in chimpanzees that recovered from hepatitis C. Blood 107: 4424–4432. 16478885 10.1182/blood-2005-09-3903 PMC1895795

[26] Richards MH, Getts MT, Podojil JR, Jin YH, Kim BS, et al (2011) Virus expanded regulatory T cells control disease severity in the Theiler's virus mouse model of MS. J Autoimmun 36: 142–154. 21273044 10.1016/j.jaut.2010.12.005 PMC3046315

[27] Belkaid Y, Rouse BT (2005) Natural regulatory T cells in infectious disease. Nat Immunol 6: 353–360. 15785761 10.1038/ni1181

[28] Cabrera R, Tu Z, Xu Y, Firpi RJ, Rosen HR, et al (2004) An immunomodulatory role for CD4(+)CD25(+) regulatory T lymphocytes in hepatitis C virus infection. Hepatology 40: 1062–1071. 15486925 10.1002/hep.20454

[29] Nilsson J, Boasso A, Velilla PA, Zhang R, Vaccari M, et al (2006) HIV-1-driven regulatory T-cell accumulation in lymphoid tissues is associated with disease progression in HIV/AIDS. Blood 108: 3808–3817. 16902147 10.1182/blood-2006-05-021576 PMC1895475

[30] Moskophidis D, Lechner F, Pircher H, Zinkernagel RM (1993) Virus persistence in acutely infected immunocompetent mice by exhaustion of antiviral cytotoxic effector T cells. Nature 362: 758–761. 8469287 10.1038/362758a0

[31] Gallimore A, Glithero A, Godkin A, Tissot AC, Pluckthun A, et al (1998) Induction and exhaustion of lymphocytic choriomeningitis virus-specific cytotoxic T lymphocytes visualized using soluble tetrameric major histocompatibility complex class I-peptide complexes. J Exp Med 187: 1383–1393. 9565631 10.1084/jem.187.9.1383 PMC2212278

[32] Wherry EJ, Ha SJ, Kaech SM, Haining WN, Sarkar S, et al (2007) Molecular signature of CD8+ T cell exhaustion during chronic viral infection. Immunity 27: 670–684. 17950003 10.1016/j.immuni.2007.09.006

[33] Zajac AJ, Blattman JN, Murali-Krishna K, Sourdive DJ, Suresh M, et al (1998) Viral immune evasion due to persistence of activated T cells without effector function. J Exp Med 188: 2205–2213. 9858507 10.1084/jem.188.12.2205 PMC2212420

[34] Virgin HW, Wherry EJ, Ahmed R (2009) Redefining chronic viral infection. Cell 138: 30–50. 19596234 10.1016/j.cell.2009.06.036

[35] Wherry EJ (2011) T cell exhaustion. Nat Immunol 12: 492–499. 21739672 10.1038/ni.2035

[36] Brooks DG, Trifilo MJ, Edelmann KH, Teyton L, McGavern DB, et al (2006) Interleukin-10 determines viral clearance or persistence in vivo. Nature medicine 12: 1301–1309. 10.1038/nm1492 PMC2535582 17041596

[37] Ejrnaes M, Filippi CM, Martinic MM, Ling EM, Togher LM, et al (2006) Resolution of a chronic viral infection after interleukin-10 receptor blockade. The Journal of experimental medicine 203: 2461–2472. 17030951 10.1084/jem.20061462 PMC2118120

[38] Tinoco R, Alcalde V, Yang Y, Sauer K, Zuniga EI (2009) Cell-intrinsic transforming growth factor-beta signaling mediates virus-specific CD8+ T cell deletion and viral persistence in vivo. Immunity 31: 145–157. 19604493 10.1016/j.immuni.2009.06.015 PMC3039716

[39] Elsaesser H, Sauer K, Brooks DG (2009) IL-21 is required to control chronic viral infection. Science 324: 1569–1572. 19423777 10.1126/science.1174182 PMC2830017

[40] Frohlich A, Kisielow J, Schmitz I, Freigang S, Shamshiev AT, et al (2009) IL-21R on T cells is critical for sustained functionality and control of chronic viral infection. Science 324: 1576–1580. 19478140 10.1126/science.1172815

[41] Yi JS, Du M, Zajac AJ (2009) A vital role for interleukin-21 in the control of a chronic viral infection. Science 324: 1572–1576. 19443735 10.1126/science.1175194 PMC2736049

[42] Boyman O, Kovar M, Rubinstein MP, Surh CD, Sprent J (2006) Selective stimulation of T cell subsets with antibody-cytokine immune complexes. Science 311: 1924–1927. 16484453 10.1126/science.1122927

[43] Pandiyan P, Zheng L, Ishihara S, Reed J, Lenardo MJ (2007) CD4+CD25+Foxp3+ regulatory T cells induce cytokine deprivation-mediated apoptosis of effector CD4+ T cells. Nat Immunol 8: 1353–1362. 17982458 10.1038/ni1536

[44] Barthlott T, Moncrieffe H, Veldhoen M, Atkins CJ, Christensen J, et al (2005) CD25+ CD4+ T cells compete with naive CD4+ T cells for IL-2 and exploit it for the induction of IL-10 production. International immunology 17: 279–288. 15684039 10.1093/intimm/dxh207

[45] Busse D, de la Rosa M, Hobiger K, Thurley K, Flossdorf M, et al (2010) Competing feedback loops shape IL-2 signaling between helper and regulatory T lymphocytes in cellular microenvironments. Proceedings of the National Academy of Sciences of the United States of America 107: 3058–3063. 20133667 10.1073/pnas.0812851107 PMC2840293

[46] McNally A, Hill GR, Sparwasser T, Thomas R, Steptoe RJ (2011) CD4+CD25+ regulatory T cells control CD8+ T-cell effector differentiation by modulating IL-2 homeostasis. Proceedings of the National Academy of Sciences of the United States of America 108: 7529–7534. 21502514 10.1073/pnas.1103782108 PMC3088596

[47] Benson A, Murray S, Divakar P, Burnaevskiy N, Pifer R, et al (2012) Microbial infection-induced expansion of effector T cells overcomes the suppressive effects of regulatory T cells via an IL-2 deprivation mechanism. Journal of immunology 188: 800–810. 10.4049/jimmunol.1100769 PMC3253229 22147768

[48] Oldenhove G, Bouladoux N, Wohlfert EA, Hall JA, Chou D, et al (2009) Decrease of Foxp3+ Treg cell number and acquisition of effector cell phenotype during lethal infection. Immunity 31: 772–786. 19896394 10.1016/j.immuni.2009.10.001 PMC2814877

[49] Attridge K, Wang CJ, Wardzinski L, Kenefeck R, Chamberlain JL, et al (2012) IL-21 inhibits T cell IL-2 production and impairs Treg homeostasis. Blood 119: 4656–4664. 22442347 10.1182/blood-2011-10-388546

[50] Wang L, Yu CR, Kim HP, Liao W, Telford WG, et al (2011) Key role for IL-21 in experimental autoimmune uveitis. Proceedings of the National Academy of Sciences of the United States of America 108: 9542–9547. 21593413 10.1073/pnas.1018182108 PMC3111309

[51] Bettelli E, Carrier Y, Gao W, Korn T, Strom TB, et al (2006) Reciprocal developmental pathways for the generation of pathogenic effector TH17 and regulatory T cells. Nature 441: 235–238. 16648838 10.1038/nature04753

[52] Veldhoen M, Hocking RJ, Atkins CJ, Locksley RM, Stockinger B (2006) TGFbeta in the context of an inflammatory cytokine milieu supports de novo differentiation of IL-17-producing T cells. Immunity 24: 179–189. 16473830 10.1016/j.immuni.2006.01.001

[53] Korn T, Bettelli E, Gao W, Awasthi A, Jager A, et al (2007) IL-21 initiates an alternative pathway to induce proinflammatory T(H)17 cells. Nature 448: 484–487. 17581588 10.1038/nature05970 PMC3805028

[54] Lahl K, Loddenkemper C, Drouin C, Freyer J, Arnason J, et al (2007) Selective depletion of Foxp3+ regulatory T cells induces a scurfy-like disease. J Exp Med 204: 57–63. 17200412 10.1084/jem.20061852 PMC2118432

[55] Boettler T, Spangenberg HC, Neumann-Haefelin C, Panther E, Urbani S, et al (2005) T cells with a CD4+CD25+ regulatory phenotype suppress in vitro proliferation of virus-specific CD8+ T cells during chronic hepatitis C virus infection. Journal of virology 79: 7860–7867. 15919940 10.1128/JVI.79.12.7860-7867.2005 PMC1143651

[56] Xu D, Fu J, Jin L, Zhang H, Zhou C, et al (2006) Circulating and liver resident CD4+CD25+ regulatory T cells actively influence the antiviral immune response and disease progression in patients with hepatitis B. Journal of immunology 177: 739–747. 10.4049/jimmunol.177.1.739 16785573

[57] Brandt L, Benfield T, Mens H, Clausen LN, Katzenstein TL, et al (2011) Low level of regulatory T cells and maintenance of balance between regulatory T cells and TH17 cells in HIV-1-infected elite controllers. Journal of acquired immune deficiency syndromes 57: 101–108. 21407087 10.1097/QAI.0b013e318215a991

[58] Punkosdy GA, Blain M, Glass DD, Lozano MM, O'Mara L, et al (2011) Regulatory T-cell expansion during chronic viral infection is dependent on endogenous retroviral superantigens. Proceedings of the National Academy of Sciences of the United States of America 108: 3677–3682. 21321220 10.1073/pnas.1100213108 PMC3048095

[59] Mempel TR, Pittet MJ, Khazaie K, Weninger W, Weissleder R, et al (2006) Regulatory T cells reversibly suppress cytotoxic T cell function independent of effector differentiation. Immunity 25: 129–141. 16860762 10.1016/j.immuni.2006.04.015

[60] Kundig TM, Schorle H, Bachmann MF, Hengartner H, Zinkernagel RM, et al (1993) Immune responses in interleukin-2-deficient mice. Science 262: 1059–1061. 8235625 10.1126/science.8235625

[61] Williams MA, Tyznik AJ, Bevan MJ (2006) Interleukin-2 signals during priming are required for secondary expansion of CD8+ memory T cells. Nature 441: 890–893. 16778891 10.1038/nature04790 PMC2776073

[62] Richter K, Perriard G, Oxenius A (2012) Reversal of chronic to resolved infection by IL-10 blockade is LCMV strain dependent. Eur J Immunol 43: 649–654. 10.1002/eji.201242887 23348876

[63] Barber DL, Wherry EJ, Masopust D, Zhu B, Allison JP, et al (2006) Restoring function in exhausted CD8 T cells during chronic viral infection. Nature 439: 682–687. 16382236 10.1038/nature04444

[64] Blackburn SD, Shin H, Haining WN, Zou T, Workman CJ, et al (2009) Coregulation of CD8+ T cell exhaustion by multiple inhibitory receptors during chronic viral infection. Nature immunology 10: 29–37. 19043418 10.1038/ni.1679 PMC2605166

[65] Jones RB, Ndhlovu LC, Barbour JD, Sheth PM, Jha AR, et al (2008) Tim-3 expression defines a novel population of dysfunctional T cells with highly elevated frequencies in progressive HIV-1 infection. The Journal of experimental medicine 205: 2763–2779. 19001139 10.1084/jem.20081398 PMC2585847

[66] Day CL, Kaufmann DE, Kiepiela P, Brown JA, Moodley ES, et al (2006) PD-1 expression on HIV-specific T cells is associated with T-cell exhaustion and disease progression. Nature 443: 350–354. 16921384 10.1038/nature05115

[67] Coquet JM, Chakravarti S, Smyth MJ, Godfrey DI (2008) Cutting edge: IL-21 is not essential for Th17 differentiation or experimental autoimmune encephalomyelitis. Journal of immunology 180: 7097–7101. 10.4049/jimmunol.180.11.7097 18490706

[68] Sonderegger I, Kisielow J, Meier R, King C, Kopf M (2008) IL-21 and IL-21R are not required for development of Th17 cells and autoimmunity in vivo. European journal of immunology 38: 1833–1838. 18546146 10.1002/eji.200838511

[69] Harker JA, Lewis GM, Mack L, Zuniga EI (2011) Late interleukin-6 escalates T follicular helper cell responses and controls a chronic viral infection. Science 334: 825–829. 21960530 10.1126/science.1208421 PMC3388900

[70] Spolski R, Wang L, Wan CK, Bonville CA, Domachowske JB, et al (2012) IL-21 promotes the pathologic immune response to pneumovirus infection. Journal of immunology 188: 1924–1932. 10.4049/jimmunol.1100767 PMC3277853 22238461

[71] Kopf M, Baumann H, Freer G, Freudenberg M, Lamers M, et al (1994) Impaired immune and acute-phase responses in interleukin-6-deficient mice. Nature 368: 339–342. 8127368 10.1038/368339a0

[72] Oxenius A, Bachmann MF, Zinkernagel RM, Hengartner H (1998) Virus-specific MHC-class II-restricted TCR-transgenic mice: effects on humoral and cellular immune responses after viral infection. European journal of immunology 28: 390–400. 9485218 10.1002/(SICI)1521-4141(199801)28:01<390::AID-IMMU390>3.0.CO;2-O

[73] Fröhlich A, Marsland BJ, Sonderegger I, Kurrer M, Hodge MR, et al (2007) IL-21 receptor signaling is integral to the development of Th2 effector responses in vivo. Blood 109: 2023–2031. 17077330 10.1182/blood-2006-05-021600

[74] Altman JD, Moss PA, Goulder PJ, Barouch DH, McHeyzer-Williams MG, et al (1996) Phenotypic analysis of antigen-specific T lymphocytes. Science 274: 94–96. 8810254 10.1126/science.274.5284.94

[75] Battegay M, Cooper S, Althage A, Bänziger J, Hengartner H, et al (1991) Quantification of lymphocytic choriomeningitis virus with an immunological focus assay in 24- or 96-well plates. J Virol Methods 191–198. 1939506 10.1016/0166-0934(91)90018-u

